# 

**Published:** 1845-12

**Authors:** 


### CONTENTS.

Page

### LIBRARY.

Anatomy of the Dental System, Human and Comparative, Translated from the French of Ph. Fr. Blandin, Surgeon of the Hotel Dieu, &c., by Robert Arthur, D. D. S.,........................ 97
A Treatise on Second Dentition, and the Natural Method of directing it; followed by a Summary of Stomatic Semeology. Translated from the French of C. F. Delabarre, by *–A–*........... 1

### JOURNAL.

ARTICLE I.—Dissertation on the Diseases of the Gums. By E. Baker, M. D., D. D. S.,.................................................... 89
ARTICLE II.—Dissertation on Tooth-Ache. By John Harris, M. D., D. D. S., 100
ARTICLE III.—Observations on the Use of Arsenic as a Remedy for Tooth-Ache, or Destroying the Lining Membrane of a Tooth. By Amos Westcott, M. D , D D. S.,.................................. 110
ARTICLE IV.—Effects of Creasote on the Economy in Health,.......... 123
ARTICLE V.—On the Application of Artificial Teeth, on Plates with Clasps. By S. M. Shepherd, D. D. S.,................................. 128
ARTICLE VI.—Letter from G. Evans,.................................. 132
ARTICLE VII.—Contributions to Operative and Mechanical Dentistry. By W. H. Elliot, D. D. S.,.......................................... 134
ARTICLE VIII.—On the Education of Dentists,............... ... ... 141
ARTICLE IX.—On Filing the Teeth. By J. Robinson, Esq., Dentist,.... 146
ARTICLE X.—Transactions of the Virginia Society of Surgeon Dentists, at their Third Annual Meeting,..................................... 149
Baltimore College of Dental Surgery. Clinical Instruction,......... .... 163

### BIBLIOGRAPHICAL NOTICES.

Manual of Diseases of the Skin, from the French of M. M. Cazenave and Schedel,............. .................................... 164

A Manual of Auscultation and Percussion. By M. Barth, Agregi to the Faculty of Medicine of Paris, &c. &c.; and M. Henry Roger, Physician to the Bureau Central of the Parisian Hospitals, &c. &c.,......... 165

A Popular Treatise on the Teeth. By Robert Arthur, D. D. S., &c.,..... 166

An Elementary Treatise on Midwifery, or Principles of Toxology and Embryology, by Alf. A. L. M. Velpeau, M. D.,........................ 166

### MISCELLANEOUS NOTICES.

Third Annual Meeting of the Virginia Society of Surgeon Dentists,....... 167
Resolutions of the American Society of Dental Surgeons, in relation to the Use of Amalgams for Filling Teeth,............. ............. 169
Arsenic as a Dental Therapeutic Agent,............................ 179
To our Subscribers in Great Britain,...... ......................... 180
Correction,...................................................... .... 181
To Correspondents,.. ................... .. ...................... 181
Museum of the Baltimore College of Dental Surgery,.................. 181
Porcelain Teeth,.................................................. 182
London Forceps,...... ........................................... 182
Convention of Dentists,........... ................................ 183
Medical Fees,...... ............................................... 184
Barley-water a powerful Diuretic,.................................. 184
J. Robinson, Esq , D. D. S., of London,............................ 184

